# Understanding the contribution of native tracheobronchial structure to lung function: CT assessment of airway morphology in never smokers

**DOI:** 10.1186/s12931-015-0181-y

**Published:** 2015-02-14

**Authors:** Alejandro A Diaz, Farbod N Rahaghi, James C Ross, Rola Harmouche, Juerg Tschirren, Raul San José Estépar, George R Washko, for the COPD Gene investigators

**Affiliations:** Division of Pulmonary and Critical Care Medicine, Department of Medicine, Brigham and Women’s Hospital, 75 Francis Street, Boston, MA 02115 USA; Surgical Planning Laboratory, Laboratory of Mathematics in Imaging, Department of Radiology, Brigham and Women’s Hospital, Harvard Medical School, Boston, MA USA; VIDA Diagnostics, Inc., Coralville, IA USA

**Keywords:** Airway wall volume, Airway lumen volume, CT, Branching generation number, Never smokers

## Abstract

**Background:**

Computed tomographic (CT) airway lumen narrowing is associated with lower lung function. Although volumetric CT measures of airways (wall volume [WV] and lumen volume [LV]) compared to cross sectional measures can more accurately reflect bronchial morphology, data of their use in never smokers is scarce. We hypothesize that native tracheobronchial tree morphology as assessed by volumetric CT metrics play a significant role in determining lung function in normal subjects. We aimed to assess the relationships between airway size, the projected branching generation number (BGN) to reach airways of <2mm lumen diameter –the site for airflow obstruction in smokers- and measures of lung function including forced expiratory volume in 1 second (FEV_1_) and forced expiratory flow between 25% and 75% of vital capacity (FEF 25–75).

**Methods:**

We assessed WV and LV of segmental and subsegmental airways from six bronchial paths as well as lung volume on CT scans from 106 never smokers. We calculated the lumen area ratio of the subsegmental to segmental airways and estimated the projected BGN to reach a <2mm-lumen-diameter airway assuming a dichotomized tracheobronchial tree model. Regression analysis was used to assess the relationships between airway size, BGN, FEF 25–75, and FEV_1_.

**Results:**

We found that in models adjusted for demographics, LV and WV of segmental and subsegmental airways were directly related to FEV_1_ (P <0.05 for all the models). In adjusted models for age, sex, race, LV and lung volume or height, the projected BGN was directly associated with FEF 25–75 and FEV_1_ (P = 0.001) where subjects with lower FEV_1_ had fewer calculated branch generations between the subsegmental bronchus and small airways. There was no association between airway lumen area ratio and lung volume.

**Conclusion:**

We conclude that in never smokers, those with smaller central airways had lower airflow and those with lower airflow had less parallel airway pathways independent of lung size. These findings suggest that variability in the structure of the tracheobronchial tree may influence the risk of developing clinically relevant smoking related airway obstruction.

**Electronic supplementary material:**

The online version of this article (doi:10.1186/s12931-015-0181-y) contains supplementary material, which is available to authorized users.

## Introduction

Expiratory airflow limitation in smokers is due to remodeling of the distal small airways and loss of lung elastic recoil because of emphysematous destruction of the lung parenchyma [1]. Computed tomographic (CT) imaging of the chest is increasingly used to assess these processes for clinical, epidemiologic, and genetic investigation [2] and there are several studies now demonstrating the association between densitometric measures of emphysema and histopathologic assessments of airspace enlargement [3]. Similar investigations of the airways are more limited. Nakano et al. [4] demonstrated that central airway wall area percent (Wall Area/Total Bronchial Area*100) was significantly correlated with lung function in smokers. A subsequent examination of lung tissue obtained from smokers at the time of surgery demonstrated that CT based morphologic assessments of the central cartilaginous airway wall predicted small airway wall dimensions [5]. These study cohorts, however, largely consist of smokers and further understanding of the airway structure may be best found in an investigation of never smoking normals. Understanding differences in structure-function relationships of the bronchial tree in this population can help to identify individuals that might be more susceptible to chronic inhalation injury such as tobacco smoke.

Recent investigations have reported highly statistically significant associations between CT cross-sectional measures of airways and lung function in never smoking normals [6,7]. Those with smaller lumen area or greater wall area percent have lower airflow in a manner similar to that observed in smokers with chronic obstructive pulmonary disease (COPD) [6,7]. To further investigate these observations we utilized volumetric airway (lumen volume [LV] and wall volume [WV]) imaging data obtained on a subset of 106 never smoking normal subjects enrolled in the COPDGene Study. Unlike cross sectional CT measures of airways, volumetric measures account for the stretch of airways with lung inflation and thus might better reflect bronchial morphology. We hypothesized that native tracheobronchial tree morphology as assessed by CT plays a significant role in determining lung function prior to the development of disease. We began by examining the relationship between extra- and intra-parenchymal airway morphology and then associated these metrics with spirometric measures of lung function. By using the lumen area ratio of daughter to parent airway generations we then sought to estimate the branching generation number (BGN) to reach <2mm-lumen-diameter airways (the airway site for airflow obstruction in smokers) [8,9]. We further hypothesized that airway size as measured as LV and WV are related to lung function and that BGN would be a predictor of lung function and possibly independent of lung size. If true, the latter would provide novel insight into the structure-function relationship of the lung.

## Methods

### Subject selection

We use data from the COPDGene Study (12 March 13 dataset), which has previously been described in detail [10]. Briefly, the goal of the study was to determine the genetic and epidemiological determinants of COPD in smokers aged 45–80 years. COPDGene also recruited a cohort of never smokers (N = 108) as a control group, which we used as the study population for this analysis. Subjects were recruited based on an eligibility questionnaire, pulmonary function test results, and chest CT scan. Subjects were considered never smokers if they answered No to the following questions: “Have you ever smoked cigarettes?”, “Have you ever been told by a physician that you had a lung disease?”, and “Have you ever had lung surgery?”. Additionally, they had to conform to a ratio of forced expiratory volume in 1 second (FEV_1_) to forced vital capacity (FVC) >=0.7 and an unremarkable CT scan. Non-smoking controls were recruited in different clinical centers across the US with the bulk of them (N = 68) in two sites (Brigham and Women’s Hospital and University of Iowa). The study was approved by the IRB at each participating center, and all patients provided written informed consent. The current analysis was approved by the Partners HealthCare Research Committee (2007P-000554).

### Clinical and physiologic assessments

Demographic and clinical data with standardized questionnaires including a modified Adult Respiratory Questionnaire were collected [10]. Spirometric measures of lung function were performed before and after the administration of albuterol according to American Thoracic Society recommendations [11]. In our analyses we used the postbronchodilator forced expiratory flow between 25% and 75% of vital capacity (FEF 25–75), FEV_1_, and FVC. The two latter spirometric measures of lung function were expressed as percent of predicted values [12].

### CT analysis

All subjects underwent volumetric CT scanning without intravenous contrast in the supine position at coached full inspiration and relaxed exhalation; analyses in this study focused on the inspiratory CT scans. The CT acquisition protocols for the three scanner brands used by COPDGene are detailed elsewhere [13]. Bronchial and lung volume measurements as detailed below were performed using the dedicated CT analysis software, Pulmonary Workstations 2 and Plus (VIDA Diagnostics, Coralville, IA, www.vidadiagnostics.com) [14] at the Imaging Center for the COPDGene study.

**Airway Measurements** Bronchial measurements were taken by trained analysts. We used airway wall volume and airway lumen volume as measures of bronchial size. We used all available data for the extra-parenchymal (right main bronchus [RMB] and left main bronchus [LMB]) and intra-parenchymal airways (segmental and subsegmental levels) collected in 6 bronchial paths: right upper lobe apical bronchus (RB1); right middle lobe lateral bronchus (RB4); right lower lobe posterior basal bronchus (RB10); left upper lobe apicoposterior bronchus (LB1 + 2); superior lingular bronchus (LB4); and left lower lobe posterior basal bronchus (LB10). These bronchi were chosen based on the consensus of COPDGene investigators and prior studies [5,15-17,13]. Airways were extracted with the region-growing method. All analyzed airways in every CT scan were visually assessed for accuracy in segmentation and labeling. Manual editing was performed to correct errors in these processes. The airway length was measured as the distance between the parent- and child-branch points by using a smoothed center line through the lumen [18]. The total airway volume, wall volume (WV), and lumen volume (LV) were calculated as airway length multiplied by the total bronchial area, wall area, and lumen area, respectively. These values were generated for all 6 bronchial paths and are presented in this study as an average for each lung and for all the 6 bronchial paths. In prior investigation, computer phantom, physical phantom, and in vivo CT reproducibility of described bronchial measures were assessed [18]. The reproducibility assessment was conducted in six bronchial trees obtained from in vivo scans of the human chest. In order to perform this assessment a reference tree with isometric cubic voxels (1 mm [3]) was built from each CT scan. For each reference tree, eight rotated instances were built and used for analysis. The branch radius, branch length, and branch volume had a mean error [95% CI of differences] of −3.39E-17 mm [−0.01–0.01], −1.23E-16 mm [−0.05–0.05], −0.05 % [−0.45–0.36], respectively [18]. Additional inter-analyst reproducibility analyses of the CT measures of airways using VIDA software were also conducted among COPDGene subjects. The inter-analyst correlation coefficient for wall thickness, lumen diameter and lumen area averaged 0.86 [19] (see Additional file 1).

We then performed a theoretical analysis for distal airways, which are out of the CT resolution, based on our CT measures of proximal airways and others’ data. We estimated the projected BGN to reach a <2mm-lumen-diameter airway (lumen area <3.14 mm [2]), which is considered a critical point for airflow obstruction in COPD [8,9]. We did this based on two assumptions: a) Weibel’s model of airway anatomy that assumes a dichotomous branching pattern [20] and b) the calculated ratio of subsegmental branch lumen area (LA) to segmental branch lumen area is preserved distally [20-22]. Note that in our investigation the 3^rd^ and 4^th^ generations from the trachea (generation 0) are considered segmental and subsegmental airways and used an average value over all branches, respectively.

$$ \mathrm{The}\kern0.5em \mathrm{project}\kern0.5em \mathrm{B}\mathrm{G}\mathrm{N}=\frac{\mathrm{In}\kern0.5em \left(3.14/\mathrm{subsegment}\kern0.5em \mathrm{lumera}\kern0.5em \mathrm{area}\right)+4}{\mathrm{In}\kern0.5em \left(\mathrm{L}\mathrm{A}\kern0.5em \mathrm{ratio}\right)} $$

where ln is the natural logarithm, LA ratio as described above, and 4 is the most distal available airway measure. We also used prior lumen diameter ratios (converted to lumen area ratios) or lumen area ratio as described by Weibel [20], Mauroy [21], and Montaudon [23] to calculate the projected BGN.

#### Lung volume measurements

Whole-lung volume on the full inspiration CT scans was expressed as a percent of predicted total lung capacity (TLC_CT_%) [24].

### Statistical analysis

Analyses were performed using SAS 9.3 (SAS Institute, Cary, NC). The relationships between extra- and intra-parenchymal airways were performed using Pearson correlations coefficients. The association between FEV_1_, airway size, and projected branching generation were assessed using regression analyses. The adjusted models included age, height, sex, and race as covariates. A P value <0.05 was considered significant.

## Results

Out of total 108 never smokers, 106 had complete data on segmental airway length in all the bronchial paths allowing calculation of wall volume and lumen volume. Subjects’ characteristics are shown in Table 1. Most of the subjects were female and Caucasians. Mean FEV_1_% predicted and FVC% predicted were 103 and 99, respectively with mean FEV_1_/FVC 0.8. We found no statistically significant differences in FEV_1_, BGN, WV, and LV between the 2 clinical centers recruiting most of the subjects and across the three most frequently used CT makes (data not shown).

**Table 1:** **Characteristics of the 106 never-smoker subjects**

**Characteristic**	**Mean ± SD or %**
Female sex (%)	69
Age (years)	62 ± 9
Caucasian race (%)	92
Height (cm)	166 ± 9
BMI (kg/m^2^)	28.2 ± 5.1
FEV_1_ (L)	2.8 ± 0.7
FEV_1_ (% predicted)	103 ± 14
FVC (L)	3.5 ± 0.9
FEF 25–75 (L)	2.87 ± 1.07
FVC (% predicted)	99 ± 12
FEV_1_/FVC ratio	0.80 ± 0.05
TLC_CT_ (L)	5.3 ± 1.2
TLC_CT_ (% predicted)	97 ± 12

### Airway dimensions

Airway dimensions are shown in Table 2. Both WV and LV decreased from extra-parenchymal airway level to segmental level to subsegmental level. The LMB had higher WV, LV, and total bronchial volume than RMB. The mean LA ratio was 0.56 (range, 0.42–0.72) and was comparable to that of Montaudon et al. [23] (Table 3) who used CT data. WV and LV of extra- and intra-parenchymal airways were significantly smaller in women than men (data not shown).

**Table 2:** **Airway measurements of never-smoker subjects**

**Airway dimension**	**Right Main Bronchus**	**Left Main Bronchus**	**All Segmental Airways**	All Subsegmental Airways*
Wall volume, mm^3^	2,981 ± 966	4,317 ± 1,214	383 ± 90	258 ± 59
Lumen volume, mm^3^	4,292 ± 1,341	6,074 ± 1,896	280 ± 84	163 ± 48
Total airway volume, mm^3^	7,272 ± 2,272	10,391 ± 3,026	662 ± 173	421 ± 106

*Subsegmental airway measures were available from 5 bronchial paths only in 3 subjects.

No subsegmental airway measures were available in 1 subject.

**Table 3:** **Lumen area ratio of subsegmental to segmental airways of never-smoker subjects**

	**Current data**	Weibel*	Mauroy**	Montaudon***
Subsegmental to Segmental Lumen Area Ratio	0.56	0.62	0.72	0.55

*This value was converted from a fixed lumen diameter ratio based on bronchial cast data [20].

**This is a theoretical value [21] and was converted from a fixed lumen diameter ratio.

***This value was calculated from CT measures of 3^rd^ and 4^th^ generation airway lumen areas provided in the Table four of the paper [23].

### Correlations between intra- and extra-parenchymal airways

Table 4 shows direct correlations between intra- and extra-parenchymal airways for both WV and LV. The correlations of LMB size or RMB size to segmental airways size ranged from 0.51 to 0.70 (P < 0.0001 for all) and were higher for LV than WV. The relationship between segmental WV of the left lung and LMB WV was slightly higher than that of its right counterpart (r = 0.61 vs. 0.51). Similar slight differences in the relationships for LV between sides were also found (r = 0.66 [left side]) vs. r = 0.58 [right side]).

**Table 4:** Pearson correlations coefficients (r)* between extra-parenchymal bronchi and intra-parenchymal segmental airways in never-smoker subjects

	**Left Main Bronchus**	**Right Lung Segmental Airways**	**Left Lung Segmental Airways**	**All Segmental Airways**
*Wall Volume*				
Right Main Bronchus	0.67	0.51	0.53	0.60
Left Main Bronchus	-	0.55	0.61	0.67
*Lumen Volume*				
Right Main Bronchus	0.84	0.58	0.64	0.68
Left Main Bronchus	-	0.59	0.66	0.70

*P < 0.0001 for all correlations.

### Associations between FEV_1_ and bronchial size

**Figure 1:**
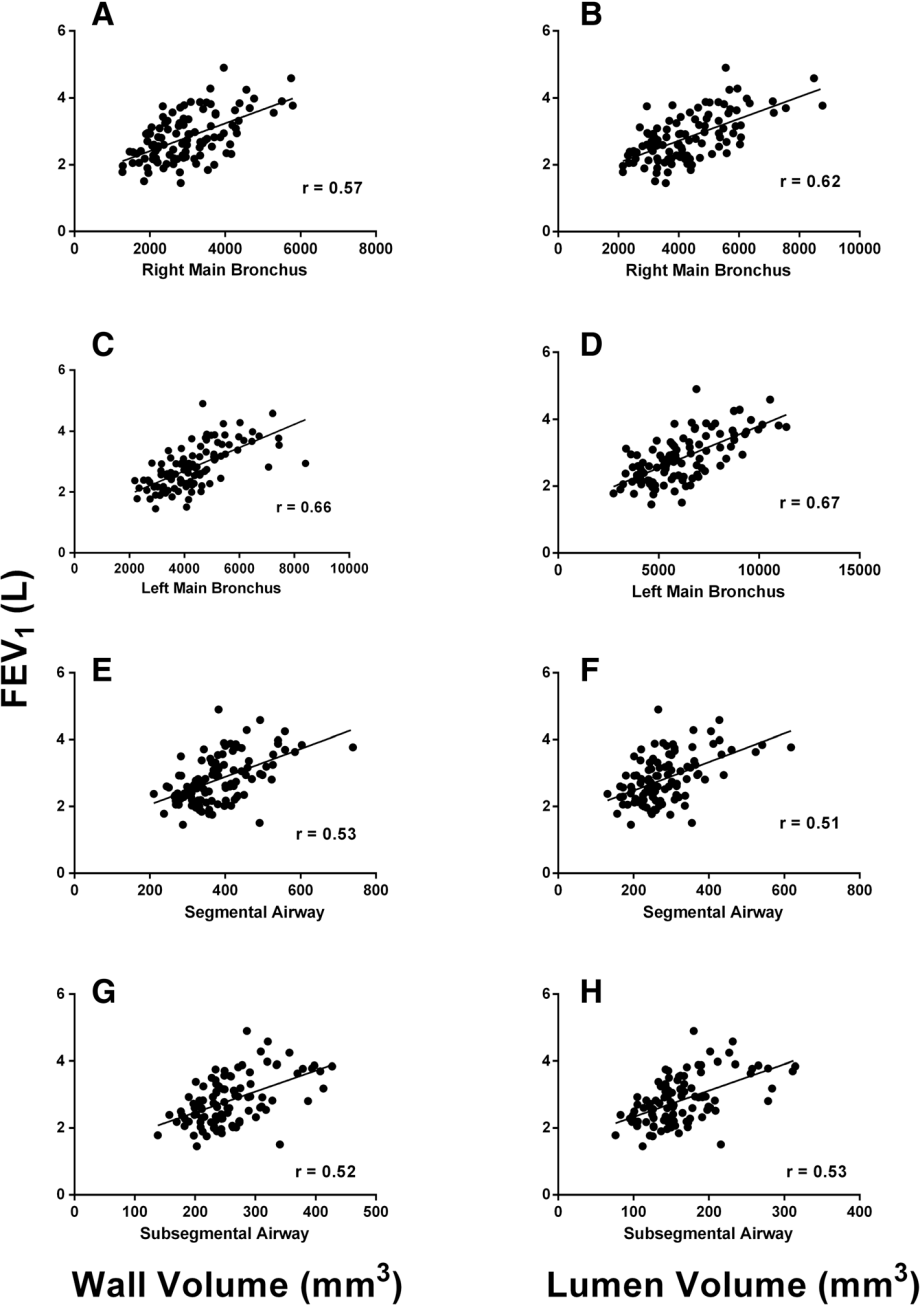
**Plot of forced expiratory volume in 1 second (FEV**_**1**_ **) as a function of airway size.** The figure shows the plot of forced expiratory volume in 1 second (FEV_1_) to wall volume (left-hand side column) and airway lumen volume (right-hand side column) in never-smoker subjects. Panels **A-D** and panels **E-H** show the plots between FEV_1_ and extra-parenchymal airways and FEV_1_ and segmental (intra-parenchymal) airways, respectively. P < 0.0001 for the correlation coefficient (r) depicted in each panel.

In univariate analysis, at extra- or intra-parenchymal airway levels there was a direct association between FEV_1_, WV, and LV (Figure 1). After adjustment for demographic variables (age, sex, race, and height) the direct relationships between FEV_1_ and LV remained statistically significant for both extra- and intra-parenchymal airways. In adjusted models, the association between FEV_1_ and WV was also significant for the RMB and subsegmental airways, on the border of significance for segmental airways, and not significant for LMB (Table 5). In order to account for the intrinsic lung volume, we replaced height by lung volume measured by CT as a covariate in the models. The results showed that both WV and LV of only subsegmental airways directly related to FEV_1_ (P = 0.03 and P = 0.048, respectively). When the multivariate models of Table 5 were performed within gender using CT lung volume as a variable for lung size, there were no significant relationships between FEV_1_ and LV or WV at any airway level (data not shown)

**Table 5:** **Multivariate regression analysis for the association between FEV**_**1**_ **(ml) and airway size at extra- and intra-parenchymal levels**

**Variable**	**Parameter (Std Error)**	**P Value**
*Airway Wall Volume (mm* ^3^ *)*		
Right Main Bronchus	0.12	0.007
Left Main Bronchus	0.06	0.17
Segmental airway	0.86	0.09
Subsegmental airway	2.18	0.003
*Airway Lumen Volume (mm* ^3^ *)*		
Right Main Bronchus	0.10	0.004
Left Main Bronchus	0.08	0.009
Segmental airway	1.16	0.03
Subsegmental airway	2.89	0.002

All models were adjusted for age, sex, race, and height.

### Associations between FEV_1_, lumen area ratio, and the projected branching generation number

**Figure 2:**
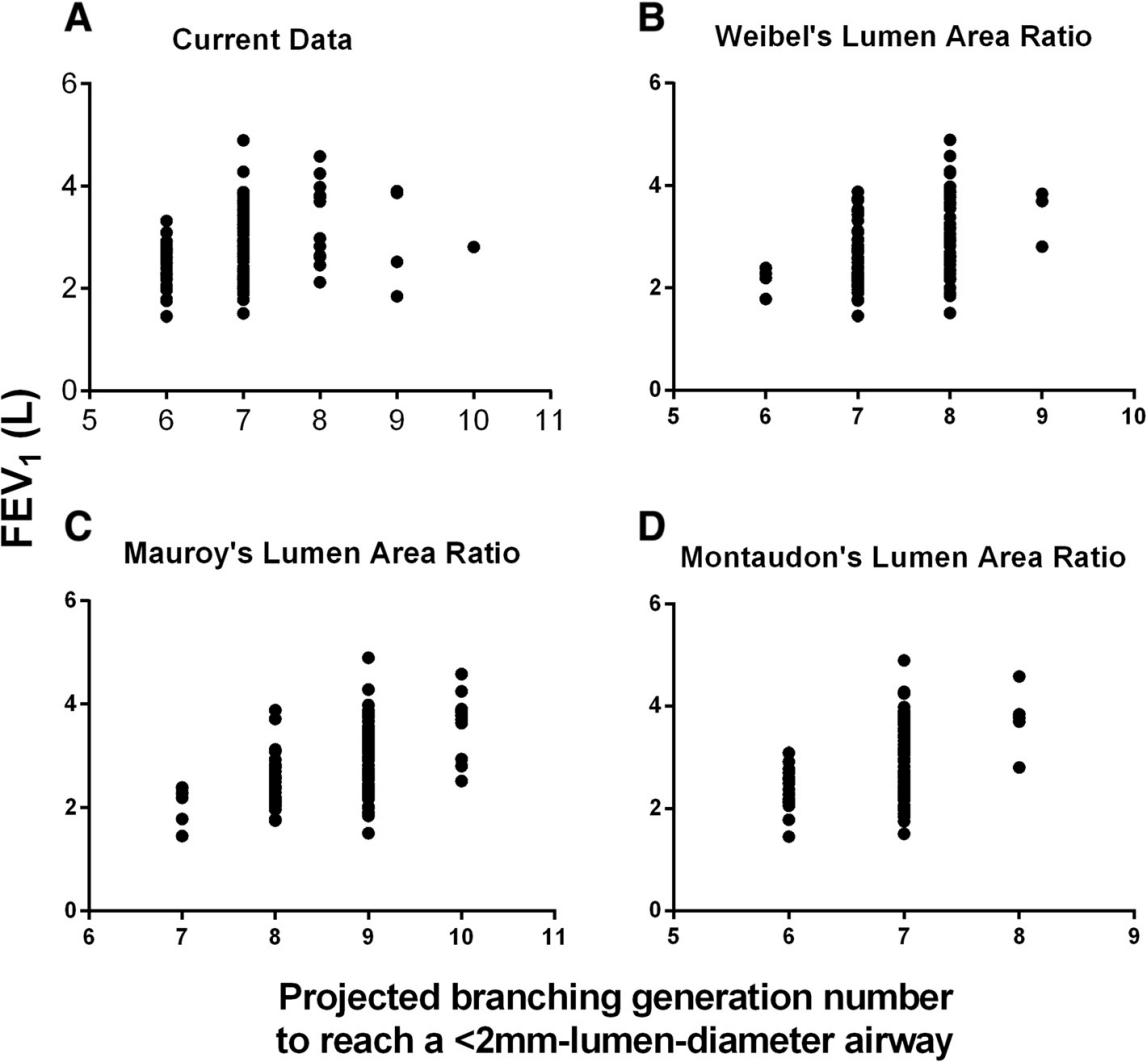
**Plot of forced expiratory volume in 1 second (FEV**_**1**_ **) as a function of the projected branching generation number.** The figures shows the plot of forced expiratory volume in 1 second (FEV_1_) to the projected branching generation number to reach <2 mm-lumen-diameter airways. Panels show the plots with the current study (Panel **A**), Weibel [20] **(B)**, Mauroy [21] **(C)**, and Montaudon’s [23] **(D)** lumen area ratios.

The LA ratio was not related to FEV_1_ (P = 0.37) nor lung volume (P = 0.72) but was to FEV_1_ % predicted (r = 0.24, P = 0.02). The mean BGN to reach a <2mm-lumen-diameter airway was 7 (range, 6–10), with 3 and 1 subjects having a BGN 9 and 10, respectively. The correlation between the projected BGN and FEV_1_ was 0.32 (P = 0.0007) and it was stronger using Weibel (r = 0.38, P < 0.0001), Mauroy (r = 0.48, P < 0.0001), or Montaudon’s (r = 0.40, P < 0.0001) lumen area ratios (Figure 2, note that the FEV_1_ tended to plateau at BGN 9 and 10). The association between our projected BGN and FEV_1_ remained significant after adjustment for demographics and LV (P = 0.001; adjusted model R [2] = 0.77) (Table 6). The standardized coefficients showed that sex, age, and height had the strongest effects on FEV_1._ In order to account for lung size, we then included the CT lung volume as a covariate instead height in multivariate models. The projected BGN remained associated with FEV_1_ (P = 0.0009; adjusted model R [2] = 0.77). Using the same above models, the projected BGN was also directly associated with FEF 25–75 (Table 6). These results persisted when analyzed by sex with the exception of the association between BGN and the FEV1 in females where the trend approach but was not statistically significant (P = 0.055).

**Table 6:** **Multivariate regression analysis for FEV**_**1**_ **and FEF 25–75 with the projected branching generation number**

	**FEV**_**1**_			**FEF 25–75**		
	**Parameter (Std Error)**	**Standardized Parameter**	**P Value**	**Parameter (Std Error)**	**Standardized Parameter**	**P Value**
Projected BGN	163 (48)	0.18	0.001	354 (114)	0.26	0.002
Segmental airway lumen volume, mm^3^	0.76 (0.50)	0.09	0.13	3.2 (1.2)	0.25	0.007
Age, yr	−31 (4)	−0.40	<0.0001	−45 (9)	−0.39	<0.0001
Female sex	−640 (104)	−0.42	<0.0001	−814 (243)	−0.35	0.001
Caucasian race	432 (134)	0.16	0.002	−254 (315)	−0.06	0.42
Height, cm	25 (5)	0.32	<0.0001	−9.7 (12.4)	−0.08	0.44

Adjusted Model R^2^ 0.77 and 0.44 for FEV_1_ and FEF 25–75, respectively.

BGN, branching generation number.

## Discussion

In this study we evaluated 106 never-smokers subjects using 3D CT measures of airways. We found highly statistically significant associations between the extra- and intra-parenchymal airways. We also report that at all selected sites in the tracheobronchial tree, the subjects’ bronchial size as measured by lumen volume and wall volume was directly related with FEV_1_. Finally, based on the assumption that the rate of reductions in lumen size between parent and daughter airway generations is uniform from central to peripheral regions of the bronchial tree, we utilized both the subsegmental airway size and rate of lumen tapering from segmental to subsegmental airway generation to calculate the projected generation at which one would find the <2mm airways. This calculated value ranged from 6 to 10 generations and was highly statistically significantly associated with the FEV_1_ even after adjustment for airway lumen volume and lung volume. This model explained 77% of the variability in FEV_1_ observed in our cohort.

Native bronchial structure measured as CT wall volume and lumen volume is related to expiratory airflow. Those subjects with smaller central airways in both univariate and in models adjusted for age, sex, race, and height have lower airflow. This also was observed at subsegmental airway level when adjusting for intrinsic lung volume. These findings are consistent with prior publications which have demonstrated a direct relationship between lumen diameter, lumen area and FEV_1_ and an indirect relationship between wall area percent and FEV_1_ [6,7]. Associations of similar direction and magnitude in smokers have been attributed to a contribution of central airway remodeling on expiratory airflow [7]. In the absence of occult airway remodeling, our findings again support conjecture that intrinsic airway structure is a significant component of what is perceived to be airway remodeling in smokers. The degree to which intrinsic lung structure and subsequent remodeling in response to chronic tobacco smoke exposure contribute to the observed relationship between CT airway data and FEV_1_ in smokers cannot, however, be directly answered with our cross sectional data.

We also found that the lumen area ratio was not related to FEV_1_ nor lung volume suggesting that a non-smoker individual’s lumen area ratio (tapering of airway size from central to peripheral regions of the lung) is independent of lung size. We further sought to further explore this finding by calculating the projected branching generation at which one would observe the <2mm airways based on that lumen area ratio. We did this under the assumption that the rate of lumen tapering beyond the 3^rd^ to 4^th^ airway generation would be constant and similar to that which we measured on CT scan proximally. As in prior studies [20,21] we also assumed that the bronchial tree was a dichotomously branching network of tubes where each generation had 2^n^ parallel pathways (where “n” is the generation). The projected BGN ranged from 6 to 10 (3 to 7 generations past the 3^rd^ generation segmental airways) where based upon our model, subjects would theoretically have between 64 and 1024 parallel pathways of similar size (for a 6^th^ generation 2mm airway 2 [6] = 64 parallel paths, for a 10^th^ generation airway 2 [10] = 1024 parallel paths). Note that these values are calculated from a single airway of origin and not meant to be an estimate of the total number of 2mm airways in the lung. After accounting for age, sex, race, segmental airway lumen volume, and lung size (height or lung volume) those subjects with a more distal or higher generation projected location of the 2mm airways had a greater FEV_1_. Further the projected BGN was associated with FEF 25–75 of vital capacity, a midflow rate measure believed to represent small airway (less than 2mm lumen diameter) patency, which supports our theoretical approach to predict distal airway tree structure.

Our data have confirmed prior findings demonstrating sex differences in the extra-parenchymal and intra-parenchymal airways [25,26]. However, when we examined the relationships between FEV_1_ and airway size and between BGN and FEV_1_ or FEF 25–75 by sex there were no differential effects of sex on these associations. Although we did not design this study to look for gender differences in airway anatomy (e.g., male and female subjects were not matched), these findings suggest that we need to more fully explore our understanding of sex differences in lung structure and how that may affect disease susceptibility.

Acknowledging the limitations of our assumptions we further sought to validate our findings by employing previously published data on the nature of airway branching obtained from both anatomic and CT based studies. Our lumen area ratio value was closer to Montaudon [23] than Weibel’s [20] and Mauroy’s [21], which may be because both our and the French study [23] used CT-based measures of airways. Using airway LA ratio data from Weibel et al. [20], Mauroy et al. [21], and Montaudon’s et al. [23], we created three additional models to calculate the projected branching generation to reach the 2mm airway. The results of each of these models were highly concordant with our findings (Figure 2) and each was observed to explain additional variance in the observed FEV_1_ of our cohort.

While the associations between lung function, airway size, and airway generation number in never smokers are straightforward, such findings may have implications for smokers and their risk of developing COPD and its clinical manifestations. Green, Mead, and Turner [27] proposed the concept of dysanaptic lung development where the bronchial tree and surrounding parenchyma develops somewhat independently. Subsequent investigations have confirmed this observation [25] and in their discussion they hypothesize that those subjects with smaller central airways may be at greater risk for airway diseases such as chronic bronchitis. In our study we found that airway lumen area ratio, a measure used to estimate the BGN, was not associated with lung size. This is consistent with Green et al.’s [27] hypothesis that lung size cannot fully predict morphology of the tracheobronchial tree. It has been proposed that for an optimized bronchial tree the diameter of the airways should decrease by a factor of 0.79 at a given branching point in order to minimize dead space volume and airflow resistance [22,28]. Based on CT measures of airways we obtained a factor of 0.56. We again believe that this difference is mostly explained by differences in method between the two studies (Murray’s model was theoretical) [22]. If our model of projected airway generation is to an extent valid, subjects with larger central airways that taper more slowly to the periphery have more airways that are of <2mm lumen diameter. They have more parallel pathways for a given lumen dimension. Since the <2mm airways have been observed to be the site of expiratory airflow obstruction in smokers [8,9], those subjects with more small airways may be more resistant to chronic noxious exposures such as tobacco smoke not because they exhibit differences in an inflammatory response but rather because they have more airways to occlude. Our findings are keeping with McDonough et al. [29] observations in lung tissue. They demonstrated that the loss of parallel pathways (small conducting airways) contribute to increased airflow resistance in subjects with COPD. We also believe that in part this may explain why subjects with COPD (vs. non-COPD) have smaller central airways measured on CT scans as it has been recently observed in large clinical studies [6,30]. Such conjecture regarding innate lung structure and susceptibility to the development of COPD and chronic bronchitis, however, requires longitudinal CT data in a cohort of smokers which is the beyond the scope of this study.

The data we present is limited by the relatively small sample size mainly made up from non-Hispanic white women, its cross sectional nature and the extent to which clinical CT scanning can quantitatively assess central airway morphology. Because of our cohort’s characteristics, generalizability of our findings is limited. We did not include 5^th^ airway generation because of potential error in overestimation of mural size (and corresponding underestimation of lumen size) would introduce noise into our model. We did, however, find clear associations between extra- and intra-parenchymal airway sizes suggesting that the airway tree is to a degree self-similar. Although the basis of our analysis is data collected between the 3^rd^ and 4^th^ generation only, we believe that this finding gives internal support to the ability of the daughter-to-parent lumen area ratio to predict the branching generation where one can find <2mm-lumen-diameter airways. Our model is also limited by the relatively simplistic assumptions that the bronchial tree is a dichotomously branching network of tubes and that the rate of tapering is constant between the central cartilaginous bronchi and distal small airways. Although some investigators believe these assumptions might be not valid because of some irregularity in the branching pattern of the bronchial tree, they have been substantiated in previous anatomic- and image-based studies [20,21] and provide a reasonable framework based on the self-similarity properties of fractal trees such as the human airways [28]. Despite these limitations our findings are supported by multivariate regression analysis which demonstrates that the projected <2mm airway generation is directly related to the FEV_1._

### Conclusion

In summary, we have demonstrated that never-smoker subjects with smaller central airways on CT scan have lower expiratory airflow. Also, our modeling suggest that independent of lung size, those subjects with a lower FEV_1_ have fewer airway generations to reach the small (<2mm-lumen-diameter) airways. These findings lead to further conjecture that variability in the structure of the tracheobronchial tree (i.e., number of distal small airways in parallel) may influence the risk of developing clinically relevant smoking related airway obstruction. Further longitudinal studies are warranted to test these findings.

## Additional file

Additional file 1:
**Quantitative CT Inter-Analyst Agreement in the COPDGene Study.**

## Abbreviations

BMI: Body Mass Index
BGN: Branching generation number
CT: Computed tomography
COPD: Chronic obstructive pulmonary disease
FEF 25–75: Forced expiratory flow between 25% and 75% of Vital capacity
FEV_1_: Forced expiratory volume in 1 second
FEV_1_/FVC: Ratio of forced expiratory volume in 1 second to forced vital capacity
FVC: Forced vital capacity
LA: Lumen area
LB1: Left upper lobe apicoposterior bronchus
LB4: Superior lingular bronchus
LB10: Left lower lobe posterior basal bronchus
LMB: Left main bronchus
LV: Lumen volume
RB1: Right upper lobe apical bronchus
RB4: Right middle lobe lateral bronchus
RB10: Right lower lobe posterior basal bronchus
RMB: Right main bronchus
SD: Standard deviation
TLC_CT_: Computed tomographic-based total lung capacity
WV: Wall volume
