# Supplementary material for: Understanding the contribution of native tracheobronchial structure to lung function: CT assessment of airway morphology in never smokers
Source: Respir Res. 2015 Feb 14;16(1):23. doi: 10.1186/s12931-015-0181-y (PMC4335784; doi:10.1186/s12931-015-0181-y)
Supplement: Additional file 1: — Quantitative CT Inter-Analyst Agreement in the COPDGene Study. [file 12931_2015_181_MOESM1_ESM.pdf]

Quantitative CT Inter-Analyst Agreement in the COPDGene Study

McKenzie A[1], Al Qaisi M[1], Williams A[1], Zach J[1], Richert D[1], Wilson C[1], Stinson D[1], Lynch DA[1], Tschirren J[2], Hoffman E[3] and the COPDGene Investigators

[1]National Jewish Health, Denver; [2]VIDA Diagnostics, Inc., Iowa City; [3]University of Iowa, Iowa City

(National Jewish Health, 1400 Jackson St, Denver, CO 80206, McKenzieA@NJHealth.org)

Introduction

This study evaluated inter-analyst agreement of quantitative computerized tomography (QCT) measures, obtained from the same CT images by two analysts.

Methods

48 CT scans, 20 from smoking control subjects (FEV_1/FVC ≥70%) and 28 from COPD subjects (FEV_1/FVC <70%), were analyzed independently by two trained research analysts using Pulmonary Workstation 2 software from VIDA Diagnostics, Inc. Differences between analysts' measures were normalized using standard deviation and evaluated using Bland-Altman methodology to determine limits of agreement more than two standard deviations from the mean. Linear regression was used to determine R^2 values for each QCT variable.

Results

Inspiratory and expiratory volume measures between analysts had an average R^2 value of 0.9829, with limits of agreement of 40.82 ± 221.47 ml and 24.06 ± 37.51 ml respectively. Density measures of the lungs and lobes had an average R^2 value of 0.9985. Limits of agreement for % inspiratory lung attenuation ≤-950 HU and % expiratory lung attenuation ≤ -856 HU were 0.01 ± 0.03% and 0.08 ± 0.11% respectively. Only 4.45% of all data evaluated were greater than 2σ from the absolute mean difference. Variation in measurements of segmental airway wall thickness, lumen diameter and inner area, was greater, with an average R^2 value of 0.7430. Limits of agreement for airway wall thickness of RB1 and RB10 were 0.01 ± 0.06 mm and 0.12 ± 0.24 mm, respectively. Limits of agreement for airway wall area of RB1 and RB10 were 0.66 ± 3.02 mm^2 and 2.74 ± 5.42 mm^2, respectively.

Conclusion

Two individual analysts, trained to the same standards and using the same analysis software, showed a high degree of reproducibility in QCT measures of emphysema and gas trapping, and a lesser degree of reproducibility for airway data.
